# Supplementary material for: Hurricane disturbance results in positive effects on tropical stream meiofauna abundance and richness
Source: PLoS One. 2026 Feb 17;21(2):e0343219. doi: 10.1371/journal.pone.0343219 (PMC12912625; doi:10.1371/journal.pone.0343219)
Supplement: Attachment — Submitted filename: Reply to reviews.docx [file pone.0343219.s002.docx]

**Response to reviewers**

**Title:** Hurricane disturbance results in positive effects on tropical stream meiofauna abundance and richness

**Authors**: Josue Santiago-Vera, Alonso Ramírez

Below we copied comments by both reviewers in black. Our responses are in blue, with line numbers referring to version 1 of the manuscript.

**Reviewer #1:**

This manuscript quantifies the effects of hurricane disturbance on meiofauna composition (abundance and richness) during 8 months pre- and post- Hurricane Irma/Maria. Changes in richness and abundances were linked to organic matter, exhibiting a trajectory towards reaching pre-hurricane levels. The study is important in the context of the existing vegetation bias in hurricane/disturbance studies and in understanding stream dynamics.

Reply: We appreciate the evaluation and comments provided by the reviewer.

The first paragraph begins with hurricane effects on flora, followed by a paragraph describing the effects on fauna. To better establish a link between hurricane effects on plants (that then cascade to faunal responses in the second paragraph), make this connection more explicit, early on in the first paragraph (instead of waiting until L60).

Reply: We edited the introduction to focus more on the effects of hurricanes on meiofauna and used the examples of plants and fauna as complementary information.

Please define resilient and resistant in L36

Reply: We added a definition.

L81: Please be more specific about why certain groups would be able to 'take advantage of the new conditions'...is this functional, behavioral, etc.

Reply: We edited this section to be more specific.

L81: Similarly, please be more specific about what changes in sediment characteristics have effects on what aspects of change in meiofauna.

Reply: We edited this section to be more specific.

L319: It's a stretch to claim that increasing richness and abundances post-hurricane indicate meiofauna are adapted to disturbance. Indeed, the following paragraph outlines other mechanisms (dispersal, environmental niche opening) unrelated to adaptation. Temper this wording (same for the use of adaptation in L353 and L415).

Reply: We changed the phrasing on this statement and others that refer to adaptation (e.g., lines 29, 319, 353, and 415 in the original submission).

L416: hurricanes have probably shaped ecosystems in Puerto Rico for thousands of years.

Reply: We replaced the term centuries for millennia.

Conclusions: New ideas were presented in this paragraph (that hurricane size floods might not be too different from non-hurricane floods; that meiofauna may have adaptations/traits to recovery from hurricane disturbance) but these ideas should also be integrated into the discussion. Then, the conclusion can expand on specific and promising lines of inquiry that future work should focus on.

Reply: We added a citation (74) that explains hurricane and non-hurricane flooding in the research area and how they are not different from each other and added the idea to the discussion section.

Figures: The resolution of the figures was low in the reviewer pdf.

Reply: We updated Figure 1 to increase the resolution. We did the same with all figures.

**Reviewer #2**:

Abstract –

The use of the term “draining” seems to imply that stream ecosystems are passive and unidirectionally moving downstream. Please replace this term.

Reply: We changed the term to located.

Throughout the manuscript there is a mixed use of names to identify the forest where the study takes place. The Luquillo Experimental Forest and El Yunque National Forest occupy the same land footprint, one is not inside the other. Please refer to the Luquillo Experimental Forest to identify your study location, and as a separate note you can state “coterminous” or “co-located” or also known as El Yunque National Forest.

Reply: We changed El Yunque National Forest to Luquillo Experimental Forest and made a note in the methodology that the Luquillo Experimental Forest is also known as El Yunque National Forest.

The coarse particulate organic matter (CPOM) noted to increase in the study streams is stated to have come from “hurricane damaged riparian forest”, yet this evidence is not presented. There have been various published studies done in tropical forests that have documented that most CPOM comes flowing from upstream of study reaches.

Reply: We edited the text and added “potentially the result of defoliation and debris from hurricane damaged of riparian forest.”

Introduction –

Please revise the order of the ideas in the Introduction. The paper begins by narrating about hurricanes and trees, not meiofauna. I suggest the authors reconsider in line with the research objectives and order of ideas presented. Share the Why, why is meiofauna important?

Reply: We edited the introduction to address the reviewer comment.

Line 36 – Please revise and clarify, is the dense wood only in the stems? Or in the whole tree?

Transitions between ideas within paragraphs should be improved, as in the case of line 39 and 40

Reply: We edited the paragraph to clarify this point.

Line 43 – The term never should be replaced to “not observed” or “have not recovered” . You can only state about the past and present, you cannot state about the future.

Reply: We removed this statement from our revised introduction.

Line 51 – There are more studies of plant communities, more studies of vegetation species. Bias would be to infer and apply what is learned about plant communities to other sets or groups of organisms. Please revise the use of “bias”.

Reply: We removed this statement from our revised introduction.

Line 58, 59 and 60 – Please revise the transitions among these ideas and the sentence structure.

Reply: We revised the introduction to address this comment.

Lines 62 to 65 –State in the initial sentence the reason why Puerto Rico offers an unique opportunity to understand hurricane impacts.

Reply: We added information to the sentence (i.e., as the island is regularly impacted by these atmospheric events). We also edited the paragraph to improve the flow of information.

Please revise the idea content and intention to present the ranges of years and scales (Caribbean versus Puerto Rico), how is it that there is no range associated to the “shortened recurrence”, 42 years with no range or error estimation?

Reply: We edited the paragraph and added a reference (38).

Line 66 – The paper cited [39] is from 2004. Is this finding still accurate?

Reply: For Puerto Rico, it is the most recent publication talking about hurricane recurrence time. We edited the sentence to improve clarity.

Line 72 and 74 – There are published studies other that [40] and done in tropical forests that have documented multi-year and pre and post hurricane stream CPOM exports and since you state that the pulse of organic matter is important to aquatic fauna, additional evidence from citations would enhance the authors arguments.

Reply: We added additional references to this statement.

Methods

Line 42 – The study cited [41] does not conduct research on the forest cover or tree species, nor does it cite where the statement of “Riparian vegetation is dominated by Dacryodes excelsa (tabonuco) and Prestoea montana (Sierra palm)” comes from. Please cite a primary reference for this statement.

Reply: We replaced the reference with a more appropriate one:

Heartsill-Scalley T, Crowl TA, Thompson J. Tree species distributions in relation to stream distance in a mid-montane wet forest, Puerto Rico. Caribbean Journal of Science. 2009;45(1):52-63.

Revise the section from line 98 to 112 – Only hurricanes get names, not tropical depressions. It is not clear why the authors are sharing the wave/depression/storm details before the categorization (and then naming) of the hurricane.

Reply: Here we are simply presenting the history of the two major hurricanes that affected our study site and provided context for our study.

Please revise to add clarity on the combination of the stated “six randomly selected pools” and the “8 samples from each tributary”. Were the “six randomly selected pools” randomized each month that sampling took place? Or just randomly selected initially for the study?

Reply: We edited the section and corrected the number of samples from 8 to 6. We collected 6 samples from each tributary for 8 months.

Line 203 – Please clarify what is meant by “monthly differences”.

Reply: We edited the sentence to clarify how the variables were selected.

RESULTS

Line 235 – Very interesting, the implications should be further developed in the Discussion section.

Reply: We added text to the discussion about this point.

Line 262 – Please remove the word “clear”, that is an interpretation that is not needed.

Reply: We removed the term clear

DISCUSSION

Line 316 – This statement seems to be contradicted with the examples presented in the second paragraph of the Discussion. Please support and revise the ideas.

Reply: We changed the term adapted to benefit, which broadens the scope and thus is not contradicted by the next paragraph.

Line 319- (and elsewhere) replace “centuries” for millennia”.

Reply: We replaced the term centuries for millenia.

Line 327 – Use of “on the other hand” seems out place here, as these are not contrasting ideas.

Reply: We changed the transition term “on the other hand” to “furthermore.”

Line 328 – Why “artificially”? Please explain the rationale.

Reply: We changed this term and improved the statement.

Line 333- There are published studies that document the stream environment altered for several years, not just several months. Please revise.

Reply: We edited the sentence to address this comment.

Please revisit the potential implications stated in the methods where there were fewer samples in the pre-hurricane data.

Reply: We edited the methods to clarify that samples were collected for eight months before and eight after the hurricanes.

Line 337- Luquillo Experimental Forest

Reply: We change El Yunque National Forest to Luquillo Experimental Forest

Line 342 – Concordance, revise to are responsible (replace “is responsible”).

Reply: We changed the term (is for are) to maintain concordance.

Line 348- explain (remove the s).

Reply: Edited.

Line 352-353 Luquillo Experimental Forest

Reply: We changed El Yunque National Forest to Luquillo Experimental Forest

Line 356 – Varies by individual ecosystem processes, not just by ecosystem and species. This

Reply: We added ecosystem processes.

Line 374 – Luquillo Experimental Forest

Reply: We changed El Yunque National Forest to Luquillo Experimental Forest

Line 401 – What was meiofauna abundance or composition related (related or correlated?) to? Shrimp abundance? This sentence contains important ideas for the manuscript and needs to be elaborated.

Reply: We specified abundance and richness of meiofauna and modified the next sentence to better explain the idea.

Line 416 – Replace century with millennia

Reply: We replaced centuries with millennia.

Line 419 – Lugo (2014) has a chapter in a book on disturbance research in Experimental Forests that describes dynamics in the LEF that includes stream response to floods. Perhaps this reference could help elaborate the idea presented here.

Reply: We added the suggested reference (below) to support arguments that stream fauna can withstand most floods.

Lugo, A. E., & Heartsill Scalley, T. (2014). Research in the Luquillo Experimental Forest has advanced understanding of tropical forests and resolved management issues. In USDA Forest Service Experimental Forests and Ranges: Research for the Long Term (pp. 435-461). New York, NY: Springer New York.

Line 422 – Is the implication that there was more hurricane damage along riparian zones than in other parts of the forest? How was it determined that the increase in organic matter that reaches the stream solely from riparian zones?

Reply: We edited the text to clarify that organic matter might have multiple sources.
